# Trimetazidine Attenuates Exhaustive Exercise-Induced Myocardial Injury in Rats via Regulation of the Nrf2/NF-κB Signaling Pathway

**DOI:** 10.3389/fphar.2019.00175

**Published:** 2019-03-05

**Authors:** Hongming Zhang, Moyan Liu, Yuyan Zhang, Xiaoyan Li

**Affiliations:** ^1^Department of Cardiology, The General Hospital of Jinan Military Area Command, Jinan, China; ^2^Department of Cardiology, The Affiliated Hospital of Guilin Medical University, Guilin, China

**Keywords:** trimetazidine, cardioprotection, exhaustive exercise, myocardial injury, apoptosis, oxidative stress

## Abstract

Exhausted exercise has been reported to cause the damage of myocardial structure and function in terms of cardiomyocyte apoptosis, oxidative stress, and energy metabolism disturbance. Trimetazidine (TMZ), as an anti-ischemic agent, has been approved to be effective in promoting myocardial energy metabolism, anti-inflammatory, and anti-oxidation. However, few studies examined the effects of TMZ on myocardial injury induced by exhausted exercise. To investigate whether TMZ could ameliorate the exhaustive exercise-induced myocardial injury and explore the underlying mechanisms, here the rat model of exhaustive exercise was induced by prolonged swimming exercise and TMZ was administrated to rats before exhaustive exercise. According to the results, we demonstrated that exhaustive exercise led to cardiomyocyte damage in rats as evidenced by elevations in cTnI and NT-proBNP levels, and decrease in CX43 expression, which was attenuated by TMZ treatment. Moreover, the administration of TMZ was found to restrain exhaustive exercise-induced oxidative stress damage by increasing GSH level, SOD and GSH-Px activities, and decreasing MDA level. Additionally, TMZ ameliorated myocardial injury by inhibiting apoptosis via reducing Bax/Bcl-2 ratio and down-regulating cleaved caspase-3, cleaved PARP, and cytochrome c levels in the myocardium of rats. Furthermore, we found that TMZ suppressed oxidative stress and cardiomyocyte apoptosis via activation of Nrf2/HO-1 and inactivation of NF-κB signaling pathways. Therefore, our study suggested that TMZ provided cardioprotection in rats after exhaustive exercise, indicating TMZ might served as a potential therapeutic drug for exhaustive exercise-induced myocardial injury.

## Introduction

Physical exercise is known to be essential to our health, and it has been well-described to decrease the risk of mortality in patients with cardiovascular and cerebrovascular diseases, respiratory disease, hypertension and diabetes mellitus (Reimers et al., 2012; Stanton et al., 2014). However, extremely heavy or excessive exercise is not always good for healthy and has adverse effect on the body, especially the heart. Thus, more and more attention has been recently focused on the damages caused by excessive exercise and its underlying mechanisms (Deaton et al., 2002; Radak et al., 2013; Ogura and Shimosawa, 2014; Olah et al., 2015). It is reported that exhaustive exercise causes myocardial injury and cardiac dysfunction, causing the increase in the risk of related cardiac diseases and conditions (Olah et al., 2015). Mounting evidence has showed that the functional impairment of exhaustive exercise-induced myocardial injury is associated with oxidative stress, cardiomyocyte apoptosis, inflammatory response, and mitochondrial dysfunction, and so on (Olah et al., 2015). However, the exact mechanism of how exhaustive exercise relates to myocardial injury remains unknown. Some studies have revealed that oxidative stress, which results from overproduction of reactive oxygen species (ROS), causing the activation of apoptosis signaling pathways, might play a crucial role in exercise-induced myocardial injury (Powers and Jackson, 2008; George et al., 2009; Muthusamy et al., 2012; Radak et al., 2013; Loboda et al., 2016). With the observation of remarkably enhanced expression of apoptosis-related regulators in damaged tissues, apoptosis is proved to be a major mechanism to explain the correlation of exhaustive exercise and followed pathological changes (Wan et al., 2017). Exhaustive exercise-induced inflammatory response has been showed to be highly elevated along with increased inflammatory cytokines after cardiac dysfunction, which put forward an alternative mechanism of exercise-induced myocardial damage (Ali and Mann, 2004; Powers and Jackson, 2008; George et al., 2009; Muthusamy et al., 2012; Loboda et al., 2016). Moreover, numerous studies have demonstrated that excessive exercise could alter mitochondrial function and structure, and thus induce permanent damages in health and disease, which provides another solution for the prevention of myocardial injury after exhaustive exercise (Huang et al., 2009; Ostojic, 2016).

Trimetazidine, a piperazine-derived anti-angina agent, is also known as 1-[2, 3, 4-trimethoxybenzyl] piperazine dihydrochloride (TMZ). It has many cardioprotective effects including reducing vascular resistance, increasing coronary and blood circulation, promoting cardiac metabolism and restoring the ability of energy production (Di Napoli and Taccardi, 2009; Tsioufis et al., 2015; Dezsi, 2016). Previous studies have demonstrated that TMZ can be effective in anti-inflammation and anti-oxidation, anti-apoptosis, improving myocardial metabolism and energy production (Martins et al., 2012; Zhou et al., 2012; Zou et al., 2017). Zhang et al. (2016) proved that the early administration of TMZ ameliorated diabetic cardiomyopathy by controlling myocardial fibrosis and cardiomyocyte apoptosis. Some studies reported that TMZ protected against cardiac ischemia-reperfusion injury by regulating miRNA-21 expression or inhibiting mitochondrial permeability transition pore (mPTP) opening (Argaud et al., 2005; Ma et al., 2016). TMZ has been evidenced to exert cardioprotection via attenuating oxidative stress, apoptosis and inflammation (Cui et al., 2017; Wan et al., 2017; He C. et al., 2018; Petrovics et al., 2018). However, data regarding the effects of pretreatment with TMZ on exhausted exercise-induced cardiac damages have been limited, and the related mechanisms are still not clear.

Here we hypothesized that TMZ might be effective to attenuate myocardial injury after exhaustive exercise. In the present study, we established an exhaustive exercise rat model by prolonged swimming exercise and rats were pretreated with TMZ before the exhaustive exercise. We aimed to gain a better understanding of the effects of TMZ on exhaustive exercise-induced myocardial injury, and its underlying cellular and molecular mechanisms.

## Materials and Methods

### Animals

This study was carried out in accordance with the recommendations of international ethical guidelines and the National Institutes of Health Guide concerning the Care and Use of Laboratory Animals. The protocol was approved by the Institutional Animal Care and Use Committee of General Hospital of Jinan Military Area Command (No. 2011-11). Adult male Sprague Dawley (SD) rats, weighting about 200 g, were purchased from Changsheng Biotechnology, Co., Ltd., Liaoning, China. The animals were allowed to access food and water freely, housed at 22 ± 1°C with a humidity of 45–55% and a 12 h light/dark cycle.

### Animal Experiments

Sprague Dawley rats were randomly divided into five groups (*n* = 12 per group): control group, TMZ group, exhausted exercise (EE) group, EE + low dose of TMZ group and EE + high dose of TMZ group. They were given by gavages with 1 ml of normal saline, 1 ml of TMZ (60 mg/kg), 1 ml of normal saline, 1 ml of TMZ (30 mg/kg) and 1 ml of TMZ (60 mg/kg) daily for 4 weeks, respectively. TMZ used in this study was purchased from Servier Pharmaceutical, Co., Ltd. (Tianjing, China). After TMZ treatment, the rats in exhausted exercise groups were initially adapted to a swimming training 20 min/day for 3 days, and then subjected to the exhausted exercise as follow: rats were forced to swim with a weight (3% of their body weight) attached to the tail until exhaustion. Exhaustion was defined as an apparent drowning over 10 s below the water surface and later no corrective reflection on the flat ground. After exhausted exercise, all rats were weighed immediately and anesthetized with pentobarbital sodium (50 mg/kg, intraperitoneal injection). Blood and left ventricular myocardial tissues were collected. One part of myocardial tissues were then fixed with 10% formaldehyde, and the other part was performed fast frozen using liquid nitrogen and stored at −80°C.

### Blood and Biochemical Analyses

The blood samples were collected from rats and stored at −80°C. The levels of alanine aminotransferase (ALT), aspartate aminotransferase (AST), creatinine, glucose, total cholesterol, high-density lipoproteincholesterol (HDL-C), low density lipoprotein cholesterol (LDL-C), triglycerides and lactate were analyzed by commercial kits (NanJing JianCheng Bioengineering Institute, China), and urea level was determined with the detection kit from Sigma (United States). The levels of malondialdehyde (MDA), superoxide dismutase (SOD), reduced glutathione (GSH) and glutathione peroxidase (GSH-Px) were detected with the detection kit from NanJing JianCheng Bioengineering Institute (Nanjing, China). The concentrations of cardiac troponin I (cTnI) and N-terminal pro-brain natriuretic peptide (NT-proBNP) in the samples were measured using ELISA kit according to the manufacturer’s instructions (Wuhan USCN Business, Co. Ltd., China).

### Hematoxylin Basic Fuchsin Picric Acid (HBFP) Staining

Hematoxylin basic fuchsin picric acid (HBFP) staining was performed in accordance with the staining kit from Beijing Leagen Biotech, Co., Ltd. (Beijing, China). Briefly, the myocardial tissues were fixed in 10% formaldehyde, embedded in paraffin and then sectioned. After deparaffinization and rehydration, the sections were stained with hematoxylin (Solarbio, China) for 3 min and immersed in ddH_2_O for 2 min. After differentiation with 1% hydrochloric acid for 3 s, they were washed with tap H_2_O for 10 min, and treated with ddH_2_O for 2 min. A basic fuchsin staining (Sangon, China) was then applied for 5 min, followed by incubation with decoloring solution for 3 min. After dehydration and coverslipping, the staining results were observed under a microscopy (DP73, Olympus, Japan).

### Real-Time Polymerase Chain Reaction (PCR)

The connexin 43 (CX43) mRNA and mitochondrial DNA (mtDNA) contents in myocardium were assessed by real-time PCR. Total RNA was extracted and then its concentration was measured. Super M-MLV reverse-transcriptase (BioTeke, Beijing, China) was used to synthesize first-strand cDNA by reverse transcription. PCR reaction system was established using SYBR Green kit (Solarbio, China), and then performed fluorescent quantitative Polymerase chain reaction using ExicyclerTM 96 (Bioneer, South Korea). Real-time PCR primers are listed below: CX43-F: 5′-CGACGACAACCAGAATGCC-3′, CX43-R: 5′-CCAACTCCACGGGAACGAA-3′; GAPDH-F: 5′-CGGCAAGTTCAACGGCACAG-3′, GAPDH-R: 5′-CGCCAGTAGACTCCACGACAT-3′. The CX43 mRNA relative content was standardized to the level of GAPDH and calculated using the 2^-ΔΔCT^ method. Total mtDNA was isolated from rat myocardium using extraction kit from Tiangen Biotech (Beijing, China) according to the manufacturer’s instructions, and the concentration was then measured. Premix Ex Taq^TM^ (Probe qPCR) from TaKaRa (Dalian, China) was applied for the real-time PCR reaction. β-actin was measured to normalize the level of mtDNA, which was calculated by the ΔCT method (ΔCT = mtDNA CT_D–loop_ – nuclearDNA CT_β–actin_).

### Western Blot

Total protein, nucleoprotein, and mitochondrial protein were isolated from myocardial tissue separately using extraction kit from Beyotime Biotechnology (Shanghai, China), and protein concentration was determined using bicinchoninic acid method (Beyotime Biotechnology, Shanghai, China). Electrophoresis was performed with 20–40 μg protein on 10% SDS polyacrylamide gels and transferred to PVDF membrane (Millipore, United States). After blocking in 5% non-fat dried milk (M/V) for 1 h, the membrane was then incubated with primary antibodies at 4°C overnight. Primary antibodies included B-cell lymphoma 2 (Bcl-2), cytochrome C, nuclear factor (erythroid-derived 2)-like 2 (Nrf2), heme oxygenase 1 (HO-1), CX43 (1:300 dilution, Wuhan Boster Biological Technology, Ltd., China), Bcl-2-associated X protein (Bax) (1:300 dilution, Sangon Biotech, Shanghai, China), nuclear factor NF-kappa-B p65 subunit (NF-κB p65) (1:500 dilution, Proteintech Group, China) and cleaved-caspase-3, cleaved-PARP, nuclear factor of kappa light polypeptide gene enhancer in B-cells inhibitor alpha (IκBα) and P-IκBα (1:1000 dilution, Cell Signaling Technology, United States). After washing with TBST, PVDF membrane was incubated with goat anti-rabbit IgG-HRP antibody (1:5000 dilution, Beyotime Biotechnology, China) at 37°C for 45 min. The result was then visualized by the enhanced chemiluminescence method and analyzed by Gel-Pro-Analyzer. The relative levels of Bcl-2, Bax, cytochrome C, Nrf2, HO-1, CX43, cleaved-caspase-3, cleaved-PARP, IκBα and P-IκBα were normalized to GAPDH (Bioss, Beijing, China), and nuclear NF-κB was standardized to Histone H3 (Sangon Biotech, Shanghai, China).

### Immunofluorescence Staining

The myocardial tissue were sectioned, deparaffinized and rehydrated, then treated with 0.1 M sodium citrate and heated in the microwave for antigen retrieval. The sections were incubated with 3% H_2_O_2_ for 15 min at room temperature (RT) and followed by a 15-min blocking goat serum (Solarbio, Beijing, China) at RT. Then they were incubated with CX43 antibody (1:100 dilution, Proteintech Group, China) at 4°C overnight. The secondary antibody at 1:200 dilution with PBS was applied to the sections for 30 min at 37°C, followed by HRP staining at 37°C for 30 min. The sections were finally treated with DAB (Solarbio, Beijing, China) and hematoxylin counter staining. After dehydration and coverslipping, the results were showed under a microscopy (DP73, Olympus, Japan).

### Electrophoretic Mobility Shift Assay (EMSA)

Total nucleoprotein was extracted from myocardial tissue using commercial extraction kit (Beyotime Biotechnology, China), according to the manufacturer’s instructions, and protein concentration was determined using bicinchoninic acid method (Beyotime Biotechnology, China). Then EMSA was conducted following the instruction from Viagene Biotech (China). Polyacrylamide gels for EMSA were prepared and the samples were diluted to 5 μg/μl with PBS. EMSA reaction was set up with 8 μl of ddH_2_O, 1.5 μl of 10× binding buffer master mix, 5 μl of protein extracts and 0.5 μl of biotin-labeled probes. After SDS-polyacrylamide gel electrophoresis, the EMSA gels were cross-linked at 10 cm under ultraviolet light for 30 min. Then they were incubated in 20 ml of blocking solution for 60 min at RT, followed by incubation with 20 ml of Streptavidin-HRP (1:750 dilution) at RT for 20 min. After washing with 20 ml of washing solution for four times, the gels were treated with 20 ml of equilibrium solution and then visualized by chemiluminescencein ECL illuminant. They were finally transferred to the dark room and performed exposure.

### TUNEL

Apoptosis was detected by TUNEL using In Situ Cell Death Detection Kit (Roche, Germany). In short, after deparaffinization, rehydration, permeabilization and blocking, myocardial tissue sections were treated with 50 μl of TUNEL reaction mixture that was prepared on ice with 10% enzyme solution and 90% label solution. The sections were incubated in that TUNEL solution at 37°C for 60 min in a humid and dark condition. After rinsing with PBS, 50 μl of converter-POD was added on the sections, which were then incubated at 37°C for 30 min. Then the sections were rinsed again with PBS and treated with 50 μl of DAB. The reaction was stopped by immersing in water as soon as the color got darker. The sections were finally stained with hematoxylin and performed coverslipping. The staining was analyzed under a microscopy (DP73, Olympus, Japan).

### Statistical Analyses

Data were presented as mean ±*SD*. Values were analyzed by one-way analysis of variance (ANOVA) followed by Bonferroni’s multiple comparison tests using GraphPad Prism 7. The value of *P* < 0.05 was considered statistically significant.

## Results

### Body Weight and Biochemical Measurements

**FIGURE 1:**
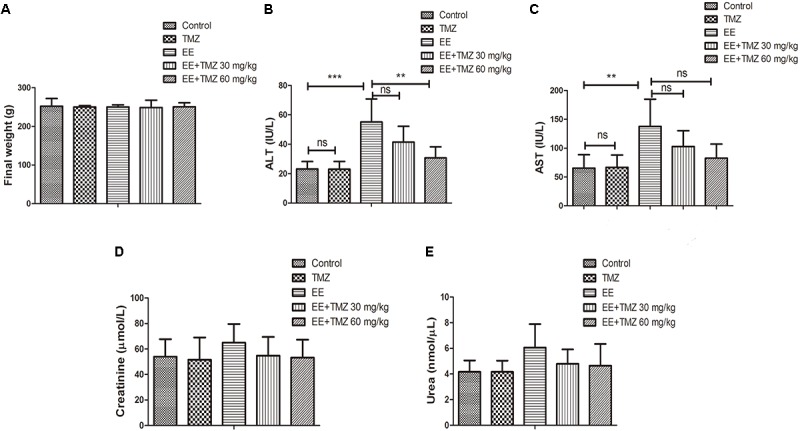
Trimetazidine (TMZ) repressed exhaustive exercise (EE)-induced changes in liver function in rats. The final body weight **(A)**, serum alanine aminotransferase (ALT) **(B)**, aspartate aminotransferase (AST) **(C)**, creatinine **(D)**, urea **(E)** levels are shown. Each value is shown as mean ±*SD* (*n* = 6). ^∗∗^*P* < 0.01, ^∗∗∗^*P* < 0.001, versus the indicated group.

**FIGURE 2:**
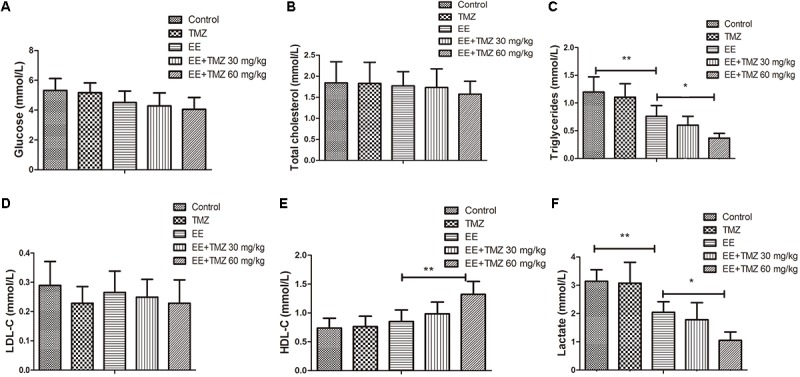
Trimetazidine inhibited EE-induced changes in metabolic parameters in rats. The serum levels of glucose **(A)**, total cholesterol **(B)**, triglycerides **(C)**, low density lipoprotein cholesterol (LDL-C) **(D)**, high density lipoprotein cholesterol (HDL-C) **(E)**, and lactate **(F)** are measured in each group. Each value is shown as mean ±*SD* (*n* = 6). ^∗^*P* < 0.05, ^∗∗^*P* < 0.01, versus the indicated group.

The final weight of the rats was measured immediately after exhaustive exercise and before anesthesia, and there was no difference in body weight among the five groups. We also analyzed the blood samples to measure the levels of ALT, AST, creatinine, urea, glucose, total cholesterol, triglycerides, LDL-C, HDL-C, and lactate. No significant difference was found in creatinine, urea, glucose, total cholesterol, and LDL-C levels (Figure 1A,D,E, 2A,B,D). For AST and ALT levels, a significant elevation was observed after exhaustive exercise and decreased levels were showed with the early administration of TMZ (Figure 1B,C). Additionally, the results showed that there was a decrease in triglycerides and lactate levels after exhaustive exercise, which were further decreased by pretreatment of TMZ (Figure 2C,F). The level of HDL-C was found to be significant higher in high dose of TMZ-treated EE rats a than that in non-treated EE rats (Figure 2E).

### Detection of Myocardium Morphology, Cardiac Function, and Myocardial Injury-Related Markers

**FIGURE 3:**
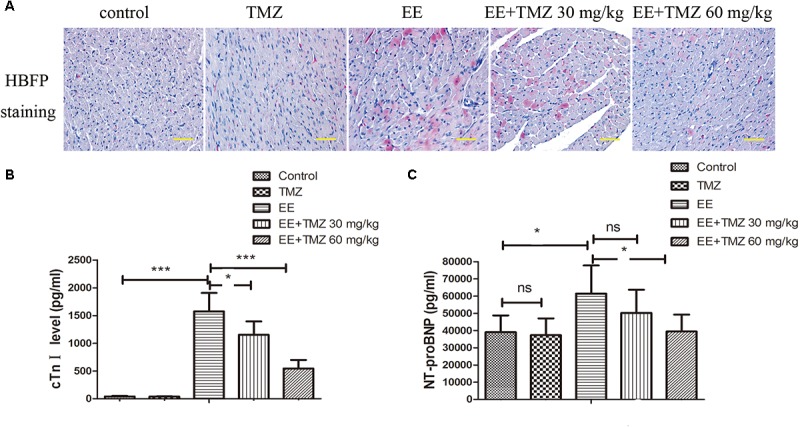
Trimetazidine alleviated EE-induced myocardial injury. **(A)** Myocardial ischemia was detected by HBFP staining. Scale bar is 50 μm. The serum cardiac troponin I (cTnI) **(B)** and N-terminal pro-brain natriuretic peptide (NT-proBNP) **(C)** levels were assessed by commercial ELISA kits. Each value is shown as mean ±*SD* (*n* = 6). ^∗^*P* < 0.05, ^∗∗∗^*P* < 0.001, versus the indicated group.

Myocardial tissues were stained using HBFP to show the pathologic changes of myocardium (Figure 3A). A mass of red staining was observed in the damaged cardiomyocytes after exhaustive exercise, while there was almost no red staining in the control rats. However, the damaged cardiomyocytes stained in red were repaired by the treatment of TMZ, and the extent of repair was more significant with high dose of TMZ treatment. Moreover, the levels of cTnI and NT-proBNP in blood samples were detected with ELISA kits (Figure 3B,C). A significant increase in cTnI and NT-proBNP levels was observed after exhaustive exercise, which were decreased by pretreatment of TMZ. As presented in Supplementary Figures S1A–G, cardiac function was detected by echocardiography. Ejection fraction and cardiac output were remarkably decreased, while left ventricular end-systolic volume was increased after exhaustive exercise. Whereas, TMZ treatment raised ejection fraction and cardiac output, and reduced left ventricular end-systolic volume.

### Observation of CX43 in Myocardium

**FIGURE 4:**
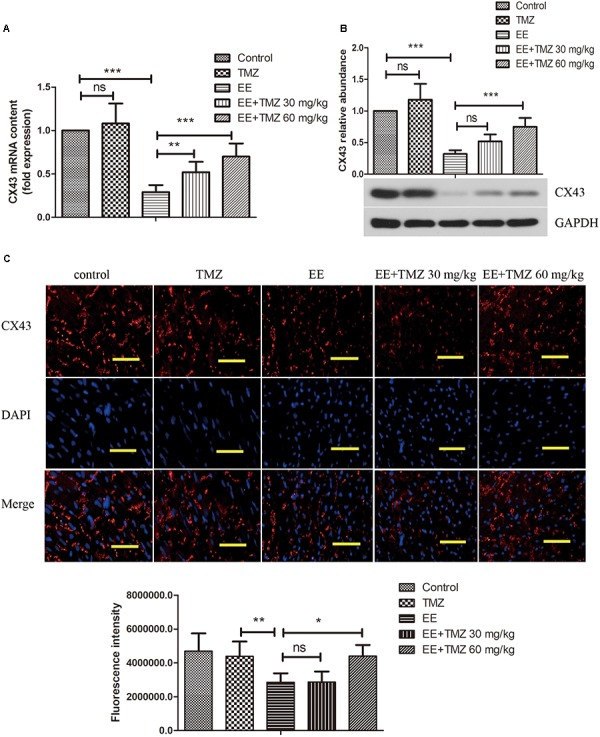
Trimetazidine suppressed EE-induced change in the expression of connexin 43 (CX43) in myocardial tissues of rats. The mRNA and protein level of CX43 was determined by real-time PCR **(A)**, and western blot **(B)**. The expression and distribution of CX43 in myocardial tissues were observed by immunofluorescence staining **(C)**. Scale bar is 50 μm. Each value is shown as mean ±*SD* (*n* = 6). ^∗^*P* < 0.05, ^∗∗^*P* < 0.01, ^∗∗∗^*P* < 0.001, versus the indicated group.

CX43 mRNA and protein levels were measured by Real-time PCR and Western blotting, respectively. According to the results, we observed a significant decrease in CX43 mRNA (Figure 4A) and CX43 protein level (Figure 4B) after exhaustive exercise compared with that in the control rats. There was a statistical difference between EE and TMZ-treated EE rats, and the levels of CX43 mRNA and protein were significantly elevated with the treatment of TMZ. In addition, we performed immunofluorescence staining to observe the expression and distribution of CX43 protein in myocardium (Figure 4C). The resultshowed that, in the control rats, the expression of gap junction protein CX43 was observed in myocardial tissue (intercalated disks), and equally distributed with clear stripe-shape. After exhaustive exercise, an obvious decrease in the intercalated disks with a dotted distribution was found in CX43, which was remarkably different from the control. However, an increase of CX43 expression was detected in the exhaustive exercised rats after TMZ treatment, which was more obvious in the rats with high dose of TMZ.

### Assessment of Oxidative Stress

**FIGURE 5:**
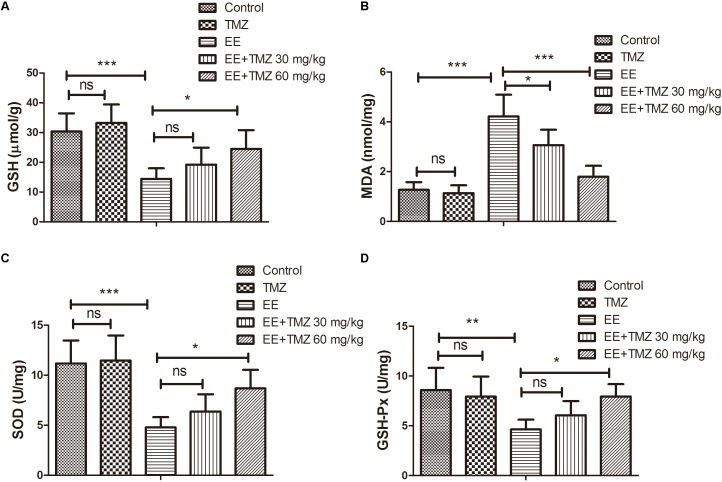
Trimetazidine restrained EE-induced oxidative stress injury in myocardial tissues of rats. The levels of reduced glutathione (GSH) **(A)**, malondialdehyde (MDA) **(B)**, and activities of superoxide dismutase (SOD) **(C)** and glutathione peroxidase (GSH-Px) **(D)** in myocardial tissues of rats were assessed. Each value is shown as mean ±*SD* (*n* = 6). ^∗^*P* < 0.05, ^∗∗^*P* < 0.01, ^∗∗∗^*P* < 0.001, versus the indicated group.

Oxidative stress was assessed by evaluating levels of GSH, MDA, SOD, and GSH-Px. The values for GSH, MDA, SOD, and GSH-Px were presented respectively in Figure 5A–D. There was a significant decrease in GSH level, SOD and GSH-Px activities, while an apparently increase in MDA level after exhaustive exercise in comparison with those in the control group. Pretreatment of TMZ was found to significantly increase the level of GSH, activities of SOD and GSH-Px, while it reduced the level of MDA.

### Observation of Cardiomyocyte Apoptosis by TUNEL

**FIGURE 6:**
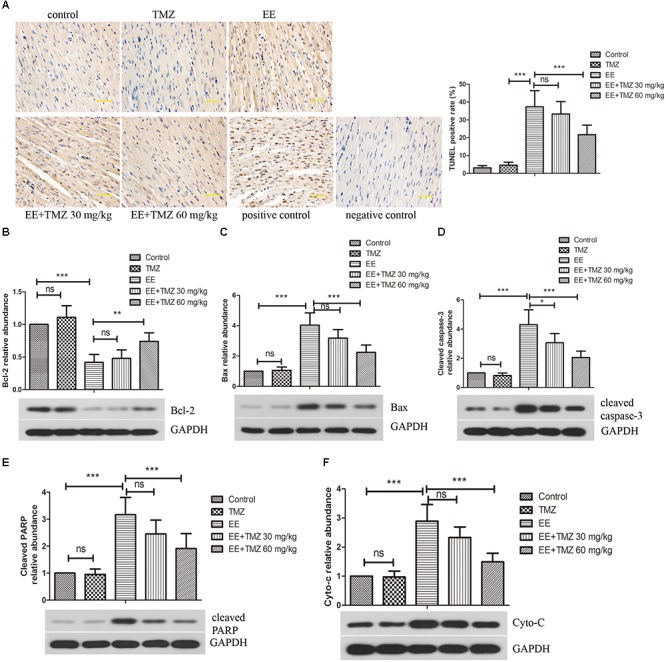
Trimetazidine inhibited EE-induced apoptosis of myocardial cells. **(A)** The apoptosis in myocardial tissues was evaluated by TUNEL staining. Scale bar is 50 μm. Western blot analysis of the levels of apoptosis-related proteins B-cell lymphoma 2 (Bcl-2) **(B)**, Bcl-2-associated X protein (Bax) **(C)**, cleaved caspase-3 **(D)**, cleaved PARP **(E)**, and cytoplasmic cytochrome complex (Cytochrome c, Cyto-C) **(F)** in myocardial tissues. GAPDH was used as a loading control. Each value is shown as mean ±*SD* (*n* = 6). ^∗^*P* < 0.05, ^∗∗^*P* < 0.01, ^∗∗∗^*P* < 0.001, versus the indicated group.

Myocardial apoptosis was evaluated by TUNEL in the five groups (Figure 6A). The results showed that no obvious apoptotic nuclei was displayed in the control rats, but there was an apparently increase in apoptosis after exhaustive exercise. In addition, pretreatment of TMZ was found to be protective to cardiomyocytes with observations of a significant decrease in apoptosis in myocardium in the EE + TMZ 30 mg/kg and EE + TMZ 60 mg/kg rats.

### Western Blot Analysis of Apoptosis-Related Proteins

The protein levels of Bcl-2, Bax, cleaved-caspase-3, cleaved-PARP and cytochrome C were analyzed by western blot (Figure 6B–F). The level of Bcl-2 was apparently decreased, while Bax, cleaved-caspase-3, cleaved-PARP and cytochrome C levels were enhanced after exhaustive exercise. Whereas, a significant increase in Bcl-2 level and decrease in Bax, cleaved-caspase-3, cleaved-PARP and cytochrome C levels were observed after the pretreatment of TMZ.

### Quantification of mtDNA in Myocardium

The content of mtDNA in myocardial tissues of rats after exhaustive exercise was determined by real-time PCR. There was no statistical difference in mtDNA content among the five groups (Table 1).

**Table 1:** Trimetazidine (TMZ) did not affect mtDNA content in myocardial tissues of rats after exhaustive exercise.

Measurement	Control	TMZ	EE	EE+TMZ 30 mg/kg	EE+TMZ 60 mg/kg
mtDNA (ΔCt)	−9.69 ± 0.08	−9.68 ± 0.10	−9.75 ± 0.08	−9.73 ± 0.08	−9.71 ± 0.07

*Data are represented as means ±SD (n = 6). TMZ, trimetazidine; EE, exhaustive exercise; mtDNA, mitochondrial DNA.*

### NF-κB and Nrf2 Signaling Pathways

**FIGURE 7:**
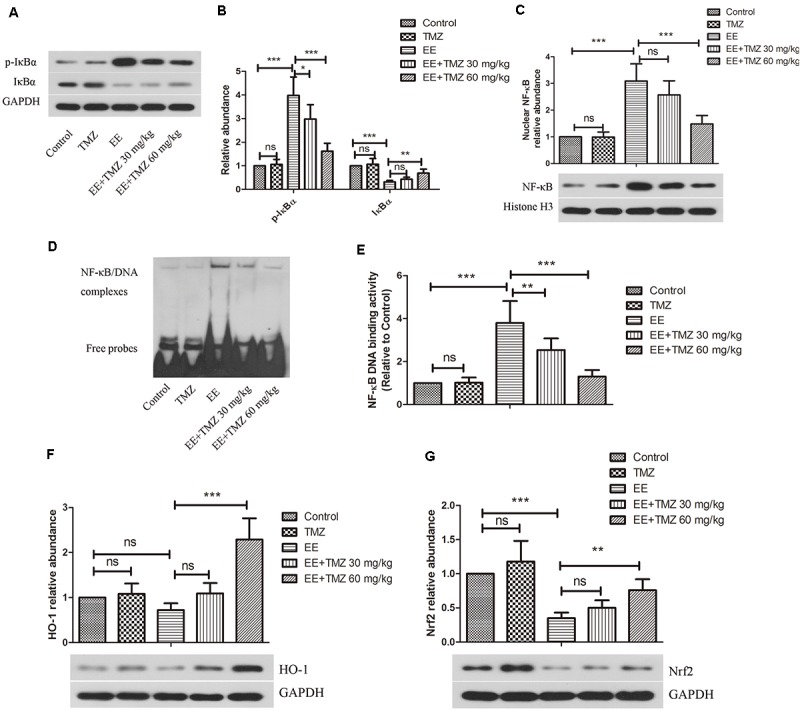
Trimetazidine restrained EE-induced activation of NF-κB and inactivation of HO-1/Nrf2 signaling pathways. **(A–C)** Western blot analysis of the protein levels of nuclear factor of kappa light polypeptide gene enhancer in B-cells inhibitor alpha (IκBα), P-IκBα, and nuclear factor kappa-light-chain-enhancer of activated B-cells (NF-κB) in myocardial tissues. GAPDH and Histone H3 were used as loading controls. **(D,E)** The DNA binding activity of NF-κB was evaluated by EMSA assay. **(F–G)** Western blot analysis of the protein levels of heme oxygenase 1 (HO-1) and nuclear factor (erythroid-derived 2)-like 2 (Nrf2) in myocardial tissues. Each value is shown as mean ±*SD* (*n* = 6). ^∗^*P* < 0.05, ^∗∗^*P* < 0.01, ^∗∗∗^*P* < 0.001, versus the indicated group.

The protein levels of IκBα, p-IκBα, NF-κB p65, HO-1, and Nrf2 in myocardial tissue were determined using western blot analysis (Figure 7A–C,F,G). An enhanced expression level of p-IκBα and a reduction in IκBα were found in the exhaustive exercised rats compared to that in the control rats, while p-IκBα level was significantly decreased and IκBα was markedly increased by TMZ pretreatment. For the nuclear NF-κB p65 expression level, there was an elevated expression in myocardium after exhausted exercise, and a significant decrease in it was detected after the pretreatment of TMZ. Besides, we performed EMSA to detect the binding activity of NF-κB p65 (Figure 7D,E), the results showed a significant higher activity in the EE rats compared with the control rats, but lowed by TMZ pretreatment. Moreover, the HO-1 and Nrf2 levels were apparently decreased in myocardium of exhaustive exercised rats, which were effectively raised by TMZ treatment.

## Discussion

Trimetazidine is a well-described antianginal drug, and it has been widely used to treat cardiovascular diseases, such as coronary heart disease, ischemia-reperfusion injury, heart failure and other cardiac diseases (Argaud et al., 2005; Martins et al., 2012; Zhou et al., 2012; Ma et al., 2016; Zhang et al., 2016; Cui et al., 2017; Zou et al., 2017; He C. et al., 2018; Petrovics et al., 2018), by restoring ischemic cardiomyocytes. In basic medical science, a large numbers of studies report that TMZ plays pivotal roles in the regulation of apoptosis, inflammatory response, and oxidative stress. For example, TMZ was evidenced to decrease cardiomyocyte apoptosis, reduce mitochondrial-dependent oxidative stress, suppress inflammatory response, and inhibit mPTP opening in ischemia-reperfusion injury (Argaud et al., 2005; Ruixing et al., 2007; Ma et al., 2016; He C. et al., 2018; Petrovics et al., 2018). However, the effects of TMZ on exhaustive exercise-induced myocardial injury remain poorly defined.

In the present study, the rat model of exhaustive exercise was induced by prolonged swimming exercise and an obvious myocardial damage was observed with increased cTnI and NT-proBNP levels, and decreased CX43 level. Pretreatment with TMZ was performed on rats to investigate the effect of TMZ on myocardial injury caused by exhaustive exercise and its underlying mechanism. According to our results, AST and ALT levels were elevated after exhaustive exercise and decreased levels were showed with the early administration of TMZ, indicating that TMZ relieved exhaustive exercise-induced liver function impairment. We also found that TMZ elevated GSH level, SOD and GSH-Px activities, and reduced MDA level, along with decreasing Bax/Bcl-2 ratio, cleaved-caspase-3, cleaved-PARP, and cytochrome C levels. The results suggested that TMZ protected against exhaustive exercise-induced oxidative stress and apoptosis. Moreover, pretreatment with TMZ was showed to decrease nuclear NF-κB level and increase Nrf2/HO-1 levels, suggesting that the cardioprotection of TMZ against exhaustive exercise-induced myocardial injury might be achieved by reducing oxidative stress and apoptosis via inactivation of NF-κB and promoting Nrf2/HO-1 signaling pathway activation.

Recent studies demonstrated that the cTnI and NT-proBNP levels, as biomarkers of myocardial damage, were increased after suffering the exhaustive exercise (Wang et al., 2015). Our results were consistent with previous studies, and showed that cTnI and NT-proBNP levels were increased by exhaustive exercise, which could be down-regulated by TMZ. It has been reported that HBFP staining is a reliable, reproducible method of demonstrating early myocardial infarction (Al-Rufaie et al., 1983). HBFP staining has been used to assess the micro-infarcted area of the myocardium in a number of studies (He W. et al., 2018; Su et al., 2018). In the present study, HBFP staining showed obvious red color in the infarct myocardium when rats were subjected to exhaustive exercise, whereas TMZ treatment made the red color lighten or disappear. CX43 is a primary protein that forms gap junctions and non-junctional hemichannels in ventricular myocardium, and it has been reported to be closely related to cardiac injury. Chang et al. (2015) demonstrated that reduced CX43 expression was observed in exhaustive exercise-induced cardiac conduction system injury. In this study, a significant decrease in CX43 expression level and distribution was observed in exhaustive exercised rats, which were largely counteracted with the pretreatment of TMZ. All these results demonstrated the myocardium damage after exhaustive swimming exercise, and treatment with TMZ presented good effects on myocardial protection.

Oxidative stress is defined as an overproduction of ROS that causes imbalance between ROS production and the capacity of the antioxidant defense system, and it consequently results in cell dysfunction and apoptosis (Powers and Jackson, 2008; Zhou et al., 2012). Oxidative stress has been evidenced to participate in exhaustive exercise-induced myocardial injury. Lipid peroxidation productions including GSH and MDA, and antioxidant enzymes including SOD and GSH-Px are considered as oxidative stress-related injury markers (Powers and Jackson, 2008; Gong et al., 2014). According to our results, impaired GSH, SOD, and GSH-Px activities and enhanced MDA level were found after exhaustive exercise. However, pretreatment with TMZ restrained the above changes in myocardium of rats. These findings suggested that TMZ could act as an effective antioxidant to counteract oxidative stress damage, which may be an important mechanism of TMZ against myocardial damage caused by exhaustive exercise.

Next, we evaluated the effect of TMZ on mtDNA. Previous studies have indicated that exhaustive exercise could lead to alteration of mtDNA, such as mtDNA^4834^ deletion in left ventricle myocardium and large-scale deletion (7052 bp) of mtDNA in acute exercise model (Huang et al., 2009; Ostojic, 2016; Zou et al., 2017). Oxidative stress and overproduction of ROS might be associated with integrity of mtDNA, which causes medium-to-large mtDNA deletions. In this study, we measured mtDNA content in myocardial tissues of rats, but there were no significant changes post-exercise or TMZ treatment. This discrepancy may due to differences in pathophysiological state of rats and inspection area. Monitoring mtDNA at specific regions might be a way to uncover alternative mechanism underlying exhaustive exercise-induced myocardial injury, which requires further examination.

It has been confirmed that the imbalance between ROS production and clearance may result in myocardial apoptosis (Fearon and Faux, 2009). So we further investigated the effect of TMZ on cardiomyocyte apoptosis. Apoptosis is a form of programmed cell death. Our results reveled that there were more TUNEL-positive apoptotic nuclei in the myocardium of rats after exhaustive exercise, whereas pretreatment with TMZ remarkably reduced the percentage of TUNEL-positive cells. Moreover, the potential mechanisms of TMZ on apoptosis were evaluated. Bcl-2 and Bax are two major proteins of the Bcl-2 family regulating apoptosis. Bax is a proapoptotic protein, while Bcl-2 performs anti-apoptosis (Cleary et al., 1986). A recent study demonstrated that TMZ protects against cardiac ischemia/reperfusion injury by augmenting the Bcl-2/Bax ratio (Ma et al., 2016). Consistent with this, the present study presented markedly increased expression of Bax and decreased expression of Bcl-2 after exhaustive exercise, indicating that the decreased Bcl-2/Bax ratio was strongly associated with myocardial injury. However, TMZ significantly inhibited exhaustive exercise-induced cardiomyocyte apoptosis by enhancing Bcl-2/Bax ratio. Additionally, cleaved caspase-3 and cleaved PARP, as important hallmarks of apoptosis, were observed to be higher in exhaustive exercise group than that in the control group, which were significantly impaired by early administration of TMZ. Cytochrome c is an essential component of electron transport chain, which also acts as an important regulator of apoptosis. The release of cytochrome c is involved in caspase-3 truncation, which results in the induction of apoptosis. Studies showed that exhaustive exercise up-regulated cytochrome c level (Ruixing et al., 2007; Huang et al., 2009; Ostojic, 2016). In consistent with their studies, we found cytochrome c was elevated in myocardium after a prolonged swimming exercise, which was significantly reduced by the pretreatment with TMZ. Our results indicated that inhibition of apoptosis was an underlying mechanism of TMZ against exhaustive exercise-induced myocardial damage.

Moreover, it is reported that mitochondrial permeability transition serves as a very important role in cardiomyocyte apoptosis following the exhaustive exercise, which is the consequence of the opening of mPTP in the mitochondrial inner membrane (Halestrap et al., 2004; Argaud et al., 2005; Ostojic, 2016). The mPTP opening is associated with DNA deletion, ATP production, calcium overload, and oxidative stress (Argaud et al., 2005; Ostojic, 2016). There are studies that showed myocardial reperfusion injury is associated with mPTP opening, which leads to inner membrane potential disturbance, respiratory chain dysfunction and release of proapoptotic mediators such as cytochrome c (Halestrap et al., 2004; Argaud et al., 2005; Huang et al., 2009). CyclosporinA was used to ameliorate cardiomyocyte apoptosis by inhibiting mPTP (Halestrap et al., 2004; Argaud et al., 2005). In these studies, TMZ was indicated to perform an effective cardioprotection against lethal ischemia-reperfusion injury via the inhibition of mPTP opening (Argaud et al., 2005). It is suggested that the inhibition of mPTP opening might be an alternative mechanism for cardioprotection, and TMZ may protect against exhaustive exercise-induced myocardial injury by intervention of mPTP opening. Based on the above studies, the effect of TMZ on exhaustive exercise-induced mPTP opening remains to be further studied.

To further investigate the protective mechanism of TMZ in exhaustive exercise-induced myocardial damage, we focused on Nrf2/ NF-κB signaling pathway. NF-κB is a transcription factor, and it controls gene expression and many inflammatory proteins, cellular growth, and apoptosis (Ali and Mann, 2004). As an early transcription factor, the activation of NF-κB does not need to be regulated by newly translated protein, and it can immediately respond to the stimulation of many different stress and conditions (Ali and Mann, 2004; Sahin et al., 2016). Thus the transcription level of NF-κB is closely related to cell damage. The activation of NF-κB is regulated by IκB kinase/NF-κB signaling pathway (Senftleben and Karin, 2002). In unstimulated cells, NF-κB is sequestered by IκB in the cytoplasm. When activated by signals, IκB is phosphorylated and degradated, and then NF-κB translocates to nucleus where it can regulate the expressions of specific genes that have DNA-binding sites for NF-κB (Surh et al., 2001; Brasier, 2006). Su et al. (2018) showed that TMZ is effective in the reduction of coronary microembolization-induced myocardial injury via inhibition of PDCD4 CME/NF-κB TNF-α signaling pathway in cardiomyocytes (Cui et al., 2017). In the present study, the phosphorylation of IκB and nuclear translocation of NF-κB were promoted by exhaustive exercise, which were suppressed by TMZ treatment. So inactivation of NF-κB signaling pathway was involved in the protective effect of TMZ against myocardial apoptosis induced by exhaustive exercise. In addition, Nrf2 is a key transcription factor of a majority of the antioxidants to defense exogenous stimulation and toxins, and responsible for the regulation of cellular redox balance, protective antioxidant and detoxification response (Ali and Mann, 2004; George et al., 2009; Muthusamy et al., 2012; Sahin et al., 2016). HO-1, as an Nrf2-dependent gene, is regulated through Nrf2 (Loboda et al., 2016). In addition, growing evidence demonstrates a complex cross-talk between Nrf2 and NF-κB [34]. The activation of Nrf2 has been found to inhibit oxidative stress as well as NF-κB signaling pathway activation [35]. The nuclear translocation of NF-κB and phosphorylation of IκB were promoted in Nrf2-silence mice [36], suggesting that the activation of Nrf2 negatively regulated NF-κB. TMZ is revealed to protect retinal ganglion cells from acute glaucoma via the Nrf2/HO-1 pathway (Wan et al., 2017). In accordance with previous studies, TMZ was demonstrated to restrain the oxidative stress damage via enhancing the decreased levels of HO-1 and Nrf2 induced by exhaustive exercise in the present study. All the above results suggested that TMZ attenuated myocardial injury after exhaustive exercise through inhibiting cell apoptosis and oxidative stress by regulating Nrf2/NF-κB signaling pathway.

The limitations of this study need to be stated. Male healthy adult rats were used in this study. So the effect of TMZ on exhaustive exercise-induced myocardial injury in different physiological (female or old) or pathological conditions cannot be explained in our study. Furthermore, inflammatory response is demonstrated to contribute to exhaustive exercise-induced myocardial injury. Besides, some studies show that TMZ treatment protects against cardiomyocyte apoptosis and myocardial injury induced by LPS via inhibition of pro-inflammatory responses (Tanoglu et al., 2015; Chen et al., 2016). However, the present study did not investigate the role of TMZ in exhaustive exercise-induced inflammatory response. All these are the direction and objects of our future research.

## Conclusion

Taken together, we demonstrate that TMZ protects against myocardial injury by attenuating exhaustive exercise-induced oxidative stress and apoptosis via activation of Nrf2/HO-1 and inactivation of NF-κB signaling pathways, which provides an insight into the use of TMZ as a potential therapeutic drug in clinical treatment and prevention as well. Both two doses (30 and 60 mg/kg) of TMZ presented beneficial effects, with higher dose more effective. Considering drug safety and efficiency, lower dosage was recommended.

## Author Contributions

HZ and XL designed and conducted the study, analyzed the data, and prepared the manuscript. ML and YZ collected and interpreted the data. All authors read and approved the final version of the manuscript.

## Conflict of Interest Statement

The authors declare that the research was conducted in the absence of any commercial or financial relationships that could be construed as a potential conflict of interest.

## Supplementary Material

FIGURE S1 TMZ repressed EE-induced changes in cardiac function in rats. The heart rate **(A)**, left ventricular end-syssolic pressure **(B)**, left ventricular end-diastolic pressure **(C)**, left ventricular end-syssolic volume **(D)**, left ventricular end-diastolic volume **(E)**, ejection fraction **(F)**, and cardiac output **(G)** are shown. Each value is shown as mean ±*SD* (*n* = 6). ^∗∗^*P* < 0.01, ^∗∗∗^*P* < 0.001, versus the indicated group.
