# Supplementary figures and images for: Trimetazidine Attenuates Exhaustive Exercise-Induced Myocardial Injury in Rats via Regulation of the Nrf2/NF-κB Signaling Pathway
Source: Front Pharmacol. 2019 Mar 5;10:175. doi: 10.3389/fphar.2019.00175 (PMC6411712; doi:10.3389/fphar.2019.00175)

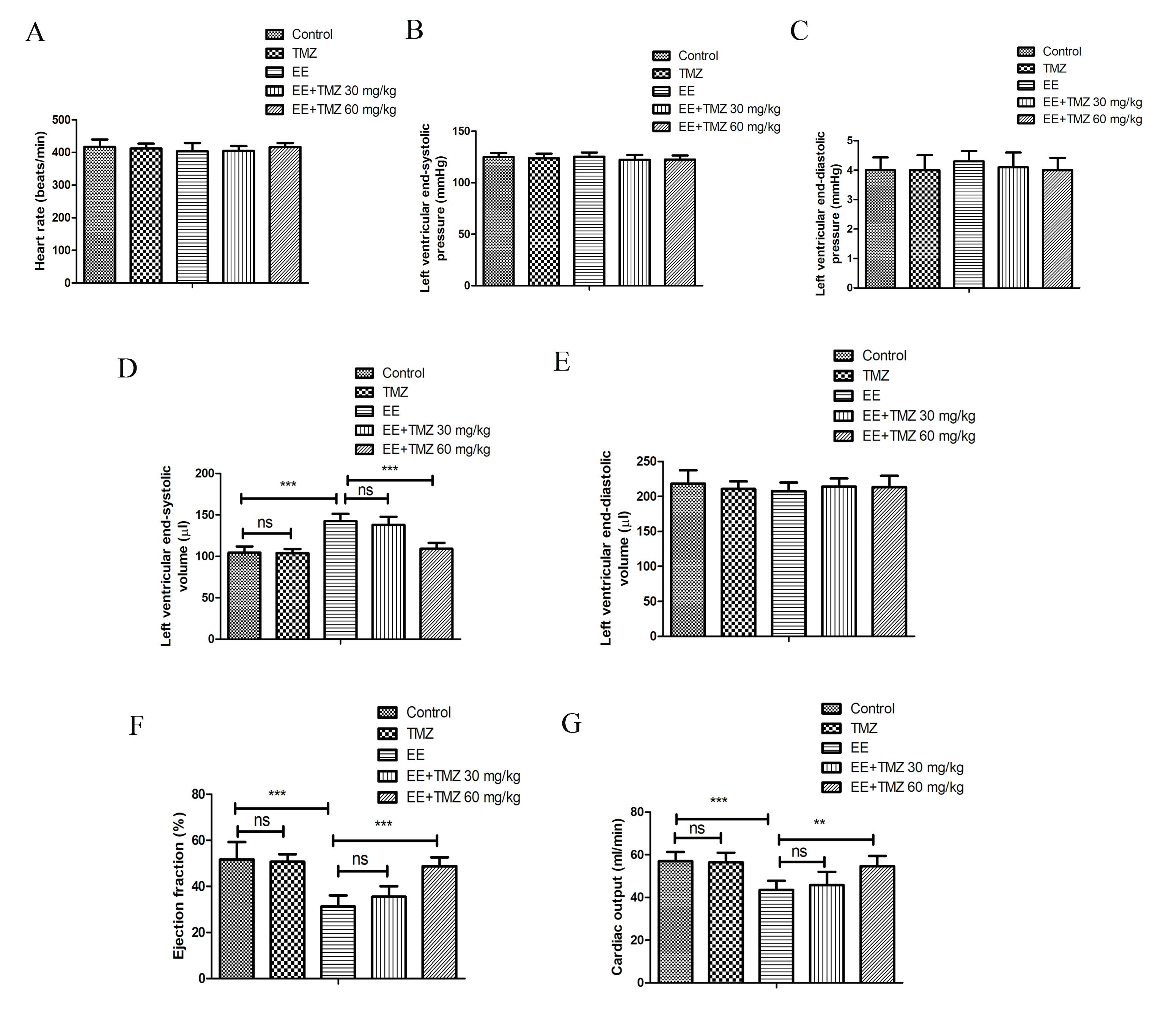

Supplement: FIGURE S1 — TMZ repressed EE-induced changes in cardiac function in rats. The heart rate (A), left ventricular end-syssolic pressure (B), left ventricular end-diastolic pressure (C), left ventricular end-syssolic volume (D), left ventricular end-diastolic volume (E), ejection fraction (F), and cardiac output (G) are shown. Each value is shown as mean ±SD (n = 6). ∗∗P < 0.01, ∗∗∗P < 0.001, versus the indicated group. [file Image_1.JPEG]
